# Identification of polyphenol composition in grape (*Vitis vinifera* cv. Bidaneh Sefid) stem using green extraction methods and LC–MS/MS analysis

**DOI:** 10.1002/fsn3.4330

**Published:** 2024-07-09

**Authors:** Shiva Beilankouhi, Amir Pourfarzad, Babak Ghanbarzadeh, Mousa Rasouli, Hamed Hamishekar

**Affiliations:** ^1^ Department of Pharmaceutical Sciences, Drug Applied Research Center University of Medical Sciences Tabriz Iran; ^2^ Department of Environmental Science Research Institute for Grapes and Raisin (RIGR) Hamedan Iran; ^3^ Faculty of Agricultural Sciences, Department of Food Science and Technology University of Guilan Rasht Iran; ^4^ Faculty of Agriculture, Department of Food Science and Technology University of Tabriz Tabriz Iran; ^5^ Faculty of Agriculture and Natural Resources, Department of Horticultural Science Engineering Imam Khomeini International University Qazvin Iran; ^6^ Drug Applied Research Center University of Medical Sciences Tabriz Iran

**Keywords:** grape stem, green extraction, LC–MS/MS, polyphenol, resveratrol

## Abstract

The utilization of grape stems, a by‐product of the grape processing industry, as a source of valuable bioactive compounds, particularly polyphenols, has gained attention in recent years. This study aimed to investigate different eco‐friendly extraction methods for obtaining polyphenols from grape (*Vitis vinifera* cv. Bidaneh Sefid) stems, focusing on green solvents and innovative techniques. Four extraction methods were tested, involving the use of water and polyethylene glycol (PEG) as green solvents, along with maceration, microwave, ultrasound, and reduced‐pressure techniques. High‐performance liquid chromatography coupled with electrospray ionization tandem mass spectrometry (HPLC–ESI‐MS/MS) was used to characterize and quantify the bioactive compounds in the extracts. A total of 29 polyphenols, including phenolic acids, flavonoids, proanthocyanidins, and stilbenes, were detected. Among the four extraction methods tested, methods 1 (water + microwave + ultrasound + atmospheric pressure) and 2 (water + microwave + ultrasound + reduced pressure) were found to be the most effective. Our study demonstrated that using water and PEG as green solvents, combined with techniques like microwave, ultrasound, and reduced pressure, effectively extracted both hydrophobic and hydrophilic compounds from the grape stems. These findings suggest that further exploration of these methods could lead to the development of value‐added products from grape stems, emphasizing the significance of green extraction techniques for the recovery of polyphenols from winemaking by‐products.

## Graphical abstract

This study aimed to investigate the use of new mixed methods, including maceration, microwave, ultrasound, and reduced‐pressure techniques, as well as water and polyethylene glycol (PEG) as green solvent for the extraction of polyphenols from grape (*Vitis vinifera* cv. Bidaneh Sefid) stems. High‐performance liquid chromatography coupled with electrospray ionization tandem mass spectrometry (HPLC–ESI‐MS/MS) was employed for the characterization and quantification of bioactive compounds. Among the four extraction methods tested, methods 1 (water + microwave + ultrasound + atmospheric pressure) and 2 (water + microwave + ultrasound + reduced pressure) emerged as the most effective, considering factors, such as cost, time, and the use of water and PEG as green solvents.

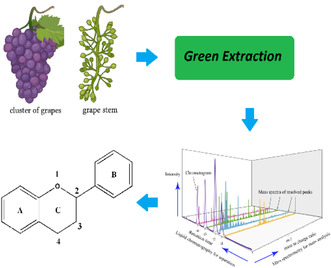

## 1 INTRODUCTION

By‐products derived from fruits are produced in large quantities all over the world. The grape agro‐industrial sector holds significant economic value (Erinle & Adewole, 2022). Grape pomace, a significant by‐product of the grape industry, consists of skins, stems, seeds, and pulp. Utilizing grape by‐products in various industrial applications, such as soil fertilization, feed, food, bioenergy, biofuel, pharmaceuticals, nutraceuticals, and cosmetic compounds, helps to minimize the environmental impact of waste disposal (Sirohi et al., 2020; Socas‐Rodríguez et al., 2021). Limited research has been conducted on the compounds found in grape stems, despite the fact that they make up 20% of the weight of harvested grapes. These stems not only contribute to environmental pollution but also serve to be utilized only for compost, animal feed, dietary fiber, and alcohol production (Maamoun, 2022).

However, the grape stem is an affordable and abundant source of vitamins, minerals, carbohydrates, and valuable plant chemical compounds. These compounds belong to the group of phenolic acids, flavonoids, tannins, and stilbenes. Interestingly, the stem contains a higher amount of these compounds compared to the pulp, leaves, and grape seeds. Additionally, the stem extract exhibited antioxidant and antiradical properties that were three times stronger than those found in the fruit flesh (Baroi et al., 2023; Constantin et al., 2024; Sorrenti et al., 2023).

Polyphenols are a group of secondary metabolites with diverse chemical structures that are produced by plants in response to physiological stress. Numerous studies have proven their anticancer, anti‐inflammatory, antimicrobial, antifungal, and immunity‐enhancing properties (Nassar, 2022). Extracting bioactive compounds from the waste of fruit juice factories and utilizing them in the food and drug industry as enrichment, dietary supplements, antioxidants, and natural preservatives will significantly contribute to the economy, industry, and health cycle (Ali et al., 2022).

Similar to the utilization of green nanocatalysts and nonedible seed oils in biodiesel synthesis (Arshad et al., 2023), our study employs environmentally friendly extraction methods to identify and characterize polyphenols in grape (*Vitis vinifera* cv. Bidaneh Sefid) stems, highlighting the importance of sustainable approaches in harnessing the potential of natural resources for various applications, such as energy production and the discovery of bioactive compounds. The most common methods used for extracting bioactive compounds from plants are stirring and solvent extraction. However, these methods result in material and time wastage. Currently, there are various techniques known as “green extraction” that have replaced these traditional methods. These new techniques are convenient, economical, and nontoxic. They not only increase the efficiency and quality of the extract but also reduce the consumption of time, solvent, and other materials. Additionally, they do not cause thermal damage to the compounds and are environmentally friendly. Green extraction is a biological alternative to the use of toxic solvents and can be applied to various types of solvents and plants (Jha & Sit, 2022). Utilizing microwaves, ultrasound, supercritical fluid, and reduced pressure in combination with solvents like water and polyethylene glycol exemplifies green extraction methods (Asl & Khajenoori, 2021).

The microwave‐assisted extraction (MAE) technique is regarded as a viable substitute for traditional methods of extracting plant extracts. The enhancement of extraction efficiency is achieved through the utilization of microwave energy and the generation of heat from the electromagnetic field. This process is facilitated by the dipole rotation of molecules, leading to a more effective extraction and the production of higher quality materials. A solvent with a higher dielectric constant possesses a superior capacity to absorb microwave energy, thereby generating uniform and adequate heat within both the solvent and solid components (AlYammahi et al., 2023; Bitwell et al., 2023).

Ultrasound‐assisted extraction (UAE) is a rapid, cost‐effective, and efficient technique for obtaining bioactive compounds. It is widely regarded as the optimal method for extracting substances that are sensitive to heat since it does not compromise their structural and functional properties. The extraction process relies on the phenomenon of cavitation, wherein ultrasound waves (20–100 KHz) generate tiny bubbles in the liquid. These bubbles then expand and disintegrate, exerting a shearing force on the surfaces. This force aids in the liberation of bioactive compounds from the source material, resulting in a higher yield and purity of the desired compounds. Additionally, the pressure surge during bubble collapse induces a shock in the plant tissue, enhancing its permeability and facilitating cell destruction. Consequently, the compounds are released from the plant into the solvent through the formation of pores in the cell walls, thereby accelerating mass transfer and increasing the overall yield. Moreover, this extraction technique can be performed at low temperatures, further preserving the integrity of the bioactive compounds (Nikolić et al., 2023; Shen et al., 2023).

The reduced‐pressure extraction (RPE) technique has become increasingly popular in recent years as an effective extraction method, particularly for heat‐sensitive compounds such as polyphenols. By lowering the extraction temperature, this method reduces damage to these compounds, leading to increased purity (Markhali & Teixeira, 2024).

To the best of our knowledge, there are no reports on the combined application of maceration, microwave, and ultrasound techniques for extracting polyphenols from grape (*Vitis vinifera* cv. Bidaneh Sefid) stems using environmentally friendly solvents. In this study, we present a novel approach that addresses this research gap by employing a unique combination of these methods and utilizing eco‐friendly solvents like water and propylene glycol, instead of nonpolar and toxic solvents commonly used in previous studies. Thus, the present study is considered the first attempt aiming to investigate and optimize green extraction methods for obtaining bioactive compounds from grape stems, an underutilized by‐product of the winemaking industry. By exploring innovative techniques and eco‐friendly solvents, we aim to contribute to the development of sustainable practices in the valorization of winemaking waste. In the following sections, we will detail our methods, present our findings, and discuss the implications of our results for future research and applications in the field of green extraction and valorization of agricultural by‐products. The extracted samples were then analyzed using liquid chromatography–tandem mass spectrometry (LC–MS/MS) to measure and identify the phenolic compounds present.

## 2 MATERIALS AND METHODS

### 2.1 Materials

Grape stalks (*Vitis vinifera* L.) were collected from gardens in Malayer city (Hamedan, Iran). The stalks were air‐dried, powdered using a laboratory mill, sieved, and stored in closed containers at room temperature until extraction. The following materials were obtained from Merck Germany and Sigma Aldrich: propylene glycol, acetonitrile, methanol, formic acid, ethanol, and high‐performance liquid chromatography (HPLC) standards.

### 2.2 Methods

#### 2.2.1 Extraction method

Four distinct extraction methods were utilized to obtain the extract from grape stems, ensuring that identical conditions were maintained for each method. A specific amount of grape stem powder was combined with deionized water and propylene glycol at a ratio of 1:10 (powder to solvent), and the mixture was then poured into glass containers with lids. To ensure a controlled environment, the containers were purged with argon and left to macerate for 72 ho at a speed of 130 rpm (revolutions per minute). Next, the mixture was subjected to ultrasound treatment in a bath operating at a power of 120 W for 25 min at 45°C. A 200 mmHg vacuum pump was connected to the ultrasound bath. After cooling, the mixture underwent microwave treatment twice, with each cycle lasting 2 min at a power of 180 W. To separate the solid particles, the mixture was then filtered through centrifugation at 8000 rpm for 20 min using filter paper. Finally, the extracts were further refined by passing them through Whatman filter paper and then dried in a vacuum oven. The resulting extracts were stored in airtight containers for subsequent analysis. Table 1 provides a summary of the specific conditions used for each extraction method (García et al., 2023; Guler, 2023; Mandal et al., 2023).

**TABLE 1:** Extraction methods conditions.

Vacuum	Ultrasound	Microwave	Solvent	Sample
−	+	+	Deionized water	1
+	+	+	Deionized water	2
−	+	+	Deionized water + propylene glycol (1:1 ratio)	3
+	+	+	Deionized water + propylene glycol (1:1 ratio)	4

#### 2.2.2 Measurement of phenolic compounds sing LC–MS/MS

Liquid chromatography coupled with mass detection is a powerful technique that can determine molecular mass, identify molecular structure, and quantify sample components. In this study, we used a liquid chromatography–tandem mass spectrometry (LC–MS/MS) system. The LC–MS/MS system consisted of an Alliance separation module 2695 system from the United States, connected to a Quattro Micro APL Triple Quadrupole LC device from Manchester, England. Chromatographic separation was performed using an Agilent ZORBAX SB‐C18 column from England. The mobile phase consisted of acetonitrile, 5% methanol, and 0.1% formic acid in water. The sample injection volume was 50 μL, and the analysis temperature was set at 40°C. Components in the sample were identified using electrospray ionization (ESI) to separate positive and negative ions. MassLynx software version 4.1 was used to control and analyze the data. Polyphenols were qualitatively identified by comparing the molecular ions and fragmentation patterns of LC–MS/MS results with existing data in the literature and a mass library for standard compounds. The concentrations were quantified using standard curves that were created with known standards. Before quantitative measurement, standard solutions were injected into the device in 10 different concentrations, and standard curves were created for each polyphenol being measured. The concentration of each polyphenol in the grape stem extract samples was then determined by comparing their peak areas to the standard curves (Cao et al., 2024; Güven et al., 2023).

### 2.3 Statistical analysis

The identification of the peaks was determined by correlating their retention times with those of the reference standards. The concentration of the analyte was determined by integrating the peaks and creating calibration curves. We used Minitab 15 software (Minitab Inc., State College, PA, USA) to perform one‐way analysis of variance (ANOVA) and test for significant differences between the mean values of different samples. All results are presented as the mean value ± standard deviation (SD) of experiments performed in triplicate. Regarding replicates, we conducted three independent extractions for each method to ensure the reliability and reproducibility of our results.

## 3 RESULTS AND DISCUSSION

In this investigation, we carried out grape stem extraction using four different green extraction methods, as outlined in Table 1. Our objective was to identify and quantify the polyphenols present in these samples. The output molecules from the LC device enter the mass detector, where they are ionized and divided into pieces using the positive and negative ESI system. This system differs for each compound. Each compound displayed a unique fragmentation pattern, allowing us to determine its molecular mass and identify the specific type of polyphenol present.

**FIGURE 1:**
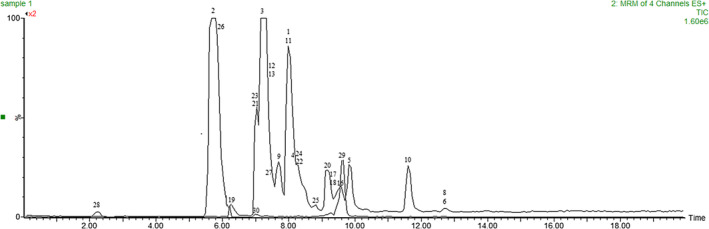
Liquid chromatography/mass spectrometry (LC/MS) chromatogram of polyphenols analyzed using ESI± identified in sample 1.

To isolate and identify the phenolic compounds in the extract, we employed the precursor/product ion transitions method. This involved obtaining the specific mass‐to‐charge ratio (m/z value) for each compound and conducting research in the existing metabolite database. We also compared the obtained data with the isotopic pattern. This comprehensive approach facilitated the successful isolation and identification of the phenolic compounds in the extract (Choi JinYoung et al., 2019). To enhance the accuracy of compound identification, we employed the Multiplex MS/MS system. The analyzed samples exhibited approximately 30 significant compounds, belonging to subgroups of phenolic acids, flavonoids, tannins, and stilbenes, through positive and negative ionization. Table 2 presents the polyphenols that were identified in the analyzed extracts using separation and instrumental analysis, based on the chromatogram peaks of the four samples under investigation. Figure 1 illustrates the chromatogram of sample 1, serving as an exemplar.

**TABLE 2:** Phenolic compounds in extracted samples (μg/g).

Peak no.	Compound	ESI^a^	Retention time (min)	[M − H] − (m/z)^b^	Sample 1	Sample 2	Sample 3	Sample 4
1	Coumaric acid	−	7.99	163 > 119	129,855 ± 558.4^b^	117,153 ± 246.0^c^	97,042 ± 329.9^d^	136,086 ± 204.1^a^
2	Gallic acid	−	5.74	169 > 125	391,496 ± 1683.4^b^	307,366 ± 645.5^c^	302,975 ± 1030.1^d^	500,854 ± 751.3^a^
3	Caffeic acid	−	7.22	179 > 135	197,001 ± 847.1^c^	172,885 ± 363.1^d^	224,467 ± 763.2^b^	444,521 ± 666.8^a^
4	Ferulic acid	−	8.28	193 > 133.9	15,180 ± 65.3^a^	15,051 ± 31.6^b^	9696 ± 33.0^d^	12,168 ± 18.3^c^
5	Trans‐Resveratrol	−	9.88	227 > 163	1121 ± 4.8^a^	916 ± 1.9^b^	905 ± 3.1^c^	694 ± 1.0^d^
6	Kaempferol 3‐galactoside	−	12.72	285 > 93	857 ± 3.7^b^	989 ± 2.1^a^	222 ± 0.8^d^	621 ± 0.9^c^
7	Luteolin	−	0	285 > 133	0^a^	0^a^	0^a^	0^a^
8	Kaempferol 3‐glucuronide	−	12.72	285 > 185	979 ± 4.2^b^	1037 ± 2.2^a^	250 ± 0.9^d^	705 ± 1.1^c^
9	Catechin	−	7.69	289 > 108.7	14,113 ± 60.7^d^	15,907 ± 33.4^c^	16,394 ± 55.7^b^	17,989 ± 27.0^a^
10	Quercetin	−	11.6	301 > 151	34,837 ± 149.8^c^	50,581 ± 106.2^a^	12,948 ± 44.0^d^	41,573 ± 62.4^b^
11	Coutaric acid	−	7.99	295 > 163	44,873 ± 193.0^a^	43,528 ± 91.4^b^	33,454 ± 113.7^c^	43,751 ± 65.6^b^
12	Caffeoyl‐tartaric acid	−	7.22	311 > 149	67,125 ± 288.6^c^	58,782 ± 123.4^d^	68,384 ± 232.5^b^	126,143 ± 189.2^a^
13	Caftaric acid	−	7.22	311 > 179	79,884 ± 343.5^a^	72,280 ± 151.8^b^	79,440 ± 270.1^a^	72,505 ± 108.8^b^
14	Myricetin	−	10.47	317 > 151	0^b^	740 ± 1.6^a^	0^b^	0^b^
15	Epicatechin gallate	−	9.17	441 > 289	0^c^	0^c^	1527 ± 5.2^a^	1331 ± 2.0^b^
16	Luteolin‐7‐glycoside	−	9.64	447 > 285	6373 ± 27.4^c^	5055 ± 10.6^d^	10,446 ± 35.5^a^	8803 ± 13.2^b^
17	Astilbin	−	9.41	449 > 151	6454 ± 27.8^a^	5045 ± 10.6^b^	4555 ± 15.5^c^	3413 ± 5.1^d^
18	Astilbin‐rhamnoside	−	9.41	449 > 303	1052 ± 4.5^a^	804 ± 1.7^b^	690 ± 2.3^c^	113 ± 0.2^d^
19	Quercetin 3‐o‐glucoside	−	6.33	477 > 151	4317 ± 18.6^c^	3089 ± 6.5^d^	6478 ± 22.0^b^	8110 ± 12.2^a^
20	Quercetin 3‐o‐glucuronide	−	9.17	477 > 331	22,544 ± 96.9^a^	20,060 ± 42.1^b^	20,066 ± 68.2^b^	16,665 ± 25.0^c^
21	Procyanidin B_1_	−	7.04	577 > 125	35,342 ± 152.0^b^	36,241 ± 76.1^a^	23,674 ± 80.5^d^	32,868 ± 49.3^c^
22	Procyanidin B_1_ 3‐o‐gallate	−	8.34	577 > 125	2956 ± 12.7^d^	5971 ± 12.5^a^	4138 ± 14.1^b^	3168 ± 4.8^c^
23	Procyanidin B_2_	−	7.04	577 > 289	46,813 ± 201.3^a^	46,806 ± 98.3^b^	31,278 ± 106.3^d^	45,182 ± 67.8^c^
24	Procyanidin B_2_ 3‐o‐gallate	−	8.34	577 > 289	2809 ± 12.1^d^	5352 ± 11.2^a^	4123 ± 14.0^c^	5037 ± 7.6^b^
25	Rutin	−	8.82	609 > 300.1	1682 ± 7.2^c^	1430 ± 3.0^d^	6272 ± 21.3^a^	4277 ± 6.4^b^
26	Procyanidin C_1_	−	5.79	865 > 125	1053 ± 4.5^b^	770 ± 1.6^c^	2190 ± 7.4^a^	0^d^
27	Procyanidin C_1_ 3‐o‐gallate	−	7.45	865 > 125	930 ± 4.0^c^	825 ± 1.7^d^	963 ± 3.3^b^	1047 ± 1.6^a^
28	ε‐Viniferin	+	2.25	445 > 215	4330 ± 18.6^b^	4573 ± 9.6^a^	1271 ± 4.3^c^	810 ± 1.2^d^
29	Cyanidin 3‐monoglucoside	+	9.59	449 > 287	45,635 ± 196.2^c^	32,971 ± 69.2^d^	64,807 ± 220.3^a^	59,698 ± 89.5^b^
30	Cyanidin 3‐sambubioside	+	7.05	581 > 287	2133 ± 9.2^b^	2240 ± 4.7^a^	1547 ± 5.3^d^	1720 ± 2.6^c^

*Note*: Data from the extracted samples are reported on a dry weight basis; Data are presented as mean ± standard deviation of three replicates; Values in rows of each compound with different letters are significantly different (*p* ≤ .05).

^**a**^ ESI, electrospray ionization;

^**b**^ Mass‐to‐charge ratio (m/z) of a molecular ion that has lost a hydrogen atom (H) and gained a negative charge.

### 3.1 Phenolic acids

**FIGURE 2:**
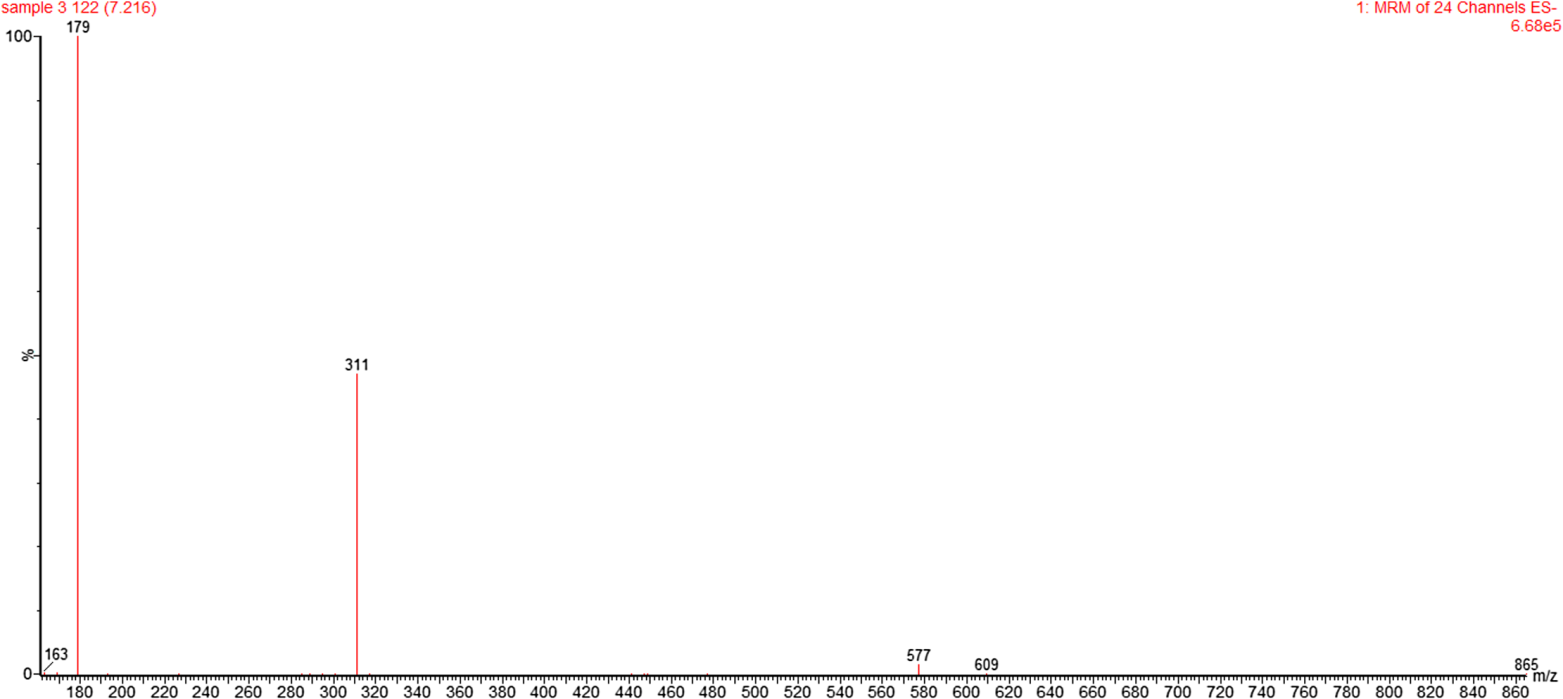
Mass spectrum of caftaric acid.

Phenolic acids are organic compounds that contain at least one aromatic ring and one or more hydroxyl groups. These molecules play various roles in the body's physiological activities, including the absorption of nutrients, enzyme activity, and protein synthesis. Recently, they have gained significant attention due to their therapeutic effects in anticancer and anti‐inflammatory drugs. An example of their impact is demonstrated by the proven ability of caffeic acid to enhance the absorption of nitric oxide and active oxygen by brain macrophages (Abd Elgadir et al., 2023; Sehrawat et al., 2022). In this study, the identification of all phenolic acids was conducted using the negative ion fraction. Coumaric acid, which had the highest concentration in the fourth sample, was assigned as peak number 1 with the main fragment (m/z) 163. Gallic acid, the phenolic compound with the highest concentration among the identified compounds, was indicated by peak number 2 with m/z 169, and sample 4 exhibited the highest amount of gallic acid. Caffeic acid, identified by peak number 3 with m/z 179, was found in the highest quantity in sample 4. A substance resembling ferulic acid, with an m/z equal to 193, was observed, although its amount was relatively lower compared to those of the previous three acids in all four samples. Coutaric acid, significant in sample 1, was assigned to peak number 11 with m/z 295. Caffeoyl‐tartaric acid and caftaric acid were detected with a signal of m/z 311 in peak numbers 12 and 13, respectively. Sample 4 exhibited the highest amount of caffeoyl‐tartaric acid, while sample 1 showed the highest amount of caftaric acid. Figure 2 illustrates the spectrometer related to caftaric acid, serving as a representative example of the specific spectrometer fingerprint for each polyphenol compound.

Our research contributes to the existing body of knowledge by offering a thorough examination of phenolic acids present in grape stem extracts. By comparing our findings with those of previous studies, we can better contextualize our results and build upon the current understanding of these compounds. Our identification of coumaric, gallic, and caffeic acids in grape stem extracts is in line with previous reports and further solidifies the existing data on these compounds. Moreover, the detection of ferulic acid and coutaric acid in our study is noteworthy, despite their relatively lower quantities. These compounds warrant further investigation to better understand their unique roles and potential contributions within grape stem extracts. By gaining a deeper understanding of the phenolic acid composition, we can more effectively harness these extracts for various applications in functional foods, supplements, or pharmaceuticals (Esparza et al., 2020, 2021). Ultimately, our work serves as a springboard for future targeted research into the bioactivity and health benefits associated with individual phenolic acids. This knowledge will be invaluable in fully realizing the potential of grape stem extracts as a source of beneficial compounds for human health and wellness.

### 3.2 Flavonoids

Flavonoids can be categorized into numerous extensive groups, including flavonols, flavanols, flavanonols, anthocyanins, flavones, chalcones, and flavanones. All of these groups can be detected through negative ionization (Ullah et al., 2020). Our study builds upon previous research by providing an in‐depth analysis of flavonoids in grape stem extracts.

#### 3.2.1 Flavonols

Flavonols encompass a range of significant compounds, such as kaempferol, quercetin, rutin, myricetin, and both free and conjugated luteolin.

Peak numbers 6 and 8 signify the existence of kaempferol galactoside and kaempferol glucuronide, respectively, with a common molecular weight of 285. Despite their limited quantity in the samples, the levels of both compounds were higher in sample 2 compared to the others. Recent research has demonstrated the analgesic, protective, antianxiety, and antidepressant properties of kaempferol (Zarei et al., 2019). Peak number 10, with a primary molecular weight of 301 and a minor weight of 151, indicates the presence of free quercetin in the extracts. Quercetin is an essential polyphenol found in the grape stem extract, and numerous studies have highlighted its antibacterial properties and effectiveness in respiratory diseases. Furthermore, quercetin and its derivatives have been proven to reduce plaque formation in blood vessels, regulate blood pressure and blood sugar levels, and possess antistress properties (Huang et al., 2020; Mitra et al., 2022). Peak numbers 19 and 20, with a molecular weight of 477, correspond to quercetin glucoside and quercetin glucuronide. Since free and bound forms of quercetin readily dissolve in water and alcohol, the second sample exhibited a high concentration of quercetin, the fourth sample contained the highest amount of quercetin glucoside, and the first sample had the most quercetin glucuronide. Myricetin, a potent anticancer compound, can induce mutagenesis in cells when present in high quantities. This compound was detected only in a small amount in the second sample, with a molecular weight of 317. Rutin, identified in peak number 25 with a molecular weight of 609, was found in higher levels in sample 3. Rutin possesses antibacterial properties, protects the liver, exhibits antitumor and anti‐inflammatory effects, aids in healing gastric ulcer, regulates the immune system, and acts as an antioxidant (Negahdari et al., 2021; Singh et al., 2022). Luteolin glycoside, responsible for the yellow color of grapes, is a valuable substance that can alleviate symptoms of multiple sclerosis by inhibiting activated leukocytes in the blood (Liang et al., 2020). It was detected at a molecular weight of 447 in peak number 16, and its concentration was highest in the third sample. However, free luteolin was generally absent in all of the extracts.

Our study expands on the current knowledge of flavonoids in grape stem extracts, highlighting the significance of individual flavonol derivatives and their potential therapeutic benefits. By investigating the full spectrum of flavonoids and their concentrations in different samples, we can understand better how these compounds contribute to the overall bioactivity of grape stem extracts and how they can be utilized in functional foods, supplements, and pharmaceuticals.

#### 3.2.2 Flavanonols

Astilbin and engeletin are classified under the group of flavanonols. Astilbin possesses specialized properties in preventing oxidation and combating diabetes (Sharma et al., 2020). Astilbin and astilbin‐rhamnoside were detected at peak numbers 17 and 18, respectively, with a mass‐to‐charge ratio (m/z) of 449. Consistent with previous studies highlighting the solubility of astilbin in methanol, ethanol, and water, we found that the concentration of these compounds was highest in the first sample, which employed extraction methods that are most efficient for extracting compounds with these solubility properties.

#### 3.2.3 Anthocyanins

Cyanidins, a specific type of anthocyanin found in grape stems, are renowned for their calming and antioxidant properties, surpassing even flavonoids. They also possess antibacterial effects and aid in reducing triglyceride and free fatty acid levels (Castilho et al., 2021). Cyanidin monoglucoside, the primary anthocyanin in grapes, was observed at peak number 29 with an m/z of 449, while cyanidin sambubioside appeared at peak number 30 with an m/z of 581, both exhibiting positive ionization fragmentation.

Compared to previous studies focusing on the overall presence of anthocyanins in grapes, we found that the highest concentration of cyanidin monoglucoside was present in the third sample, while cyanidin sambubioside was most abundant in the second sample. This finding highlights the importance of investigating individual anthocyanin compounds in grape stem extracts, as their relative concentrations may vary depending on the extraction method and grape cultivar.

### 3.3 Tannins (polymeric proanthocyanidins)

#### 3.3.1 Catechin

An aromatic phenolic compound with the molecular formula C_6_H_5_OH, it exhibits various beneficial effects on human health. It has been found to effectively eliminate free radicals, lower levels of harmful cholesterol while increasing the levels of high‐density lipoprotein (HDL), enhance fat metabolism in the liver, promote blood thinning, and combat viral and bacterial infections. Furthermore, catechin has demonstrated the ability to alleviate allergy symptoms and act as an oral disinfectant (Ozato et al., 2022). Notably, a study has provided evidence of catechin's positive impact on reducing breast cancer cells, with proven efficacy at a concentration of 30 μg (Almatroodi et al., 2020). In the present investigation, catechin was identified at an m/z equal to 289 and observed in peak number 9.

In the context of our investigation, the solubility of catechin in alcohol was considered, along with the utilization of vacuum to minimize thermal damage. This approach resulted in an increase in catechin concentration from sample 1 to 4, suggesting that optimizing extraction methods can enhance the yield of this valuable compound from grape stem extracts.

#### 3.3.2 Epicatechin gallate

A potent compound possessing both antioxidant and antibacterial properties, it exhibits remarkable efficacy in enhancing the immune and nervous systems of the human body (Payne et al., 2022).

Although epicatechin gallate is primarily found in green tea, it is also present in grapes in limited quantities. Our study detected trace amounts of this compound in samples 3 and 4, attributable to its solubility in organic solvents and alcohol. The presence of m/z 441 in peak number 15 is indicative of the presence of this compound. Our findings align with those of previous studies highlighting the potential health benefits of epicatechin gallate. Given its therapeutic properties and the fact that it is present in trace amounts in grapes, optimizing extraction methods to maximize its yield from grape stem extracts is of interest.

#### 3.3.3 Procyanidin B_1_, procyanidin B_2_, and procyanidin

C_1_ are significant phenolic compounds found in grape stem and core. These compounds have a complex structure and can exist in both free and connected forms. One of the key physicochemical characteristics of procyanidins is their ability to form strong bonds with proteins (Liu et al., 2021). Procyanidins, particularly B_2_, possess numerous health‐promoting properties. These include reducing the risk of cardiovascular diseases and diabetes, enhancing resistance against the HIV (human immunodeficiency virus), acting as a potent absorber of superoxide and hydroxyl radicals in plasma, and reducing oxidative stress and inflammation. Procyanidins are often consumed as nutritional supplements without any adverse effects (Dasiman et al., 2022). In the analyzed samples, peak numbers 21, 22, 23, and 24 with a parent ion mass‐to‐charge ratio (m/z) of 577 and daughter ions at 125 and 289 indicate the presence of procyanidins B1 and B2. The second sample exhibited the highest level of these compounds under the curve. Peak numbers 26 and 27, with a main m/z of 865 and a minor m/z of 125, confirm the presence of free and polymeric procyanidin C_1_. Sample 3 demonstrated the highest amount of procyanidin C_1_.

### 3.4 Stilbenes

#### 3.4.1 Trans‐resveratrol

Let's move on to 4‐stilbenes, specifically trans‐resveratrol, which is a significant compound found in grapes. Grapes are considered one of the most important and limited sources of resveratrol, and resveratrol can be extracted in large quantities from various parts of the grape. This potent antioxidant is soluble in water and alcohols. Resveratrol has shown anticancer properties, particularly against prostate cancer and colon cancer. It also has antiradical and antimicrobial effects, and provides cardiovascular protection by preventing plaque accumulation (Islam et al., 2022; Yang et al., 2021).

#### 3.4.2 Viniferin

Viniferin, a resveratrol dehydrodimer, has the chemical formula C_28_H_22_O_6_. It exists in different forms, including ε‐viniferin, α‐viniferin, and β‐viniferin. Grape products are the primary source of these compounds, with a higher concentration found in the stems compared to the rest of the grape. Various types of grapes contain viniferins. These compounds, known as antioxidants, have been found to have potential in preventing obesity (Gómez‐Zorita et al., 2023). In our study, we detected the presence of epsilon (ε‐) viniferin at 445 and peak number 28 through positive molecular ionization. The second sample exhibited a larger area under the curve, indicating a higher concentration of epsilon viniferin compared to the other samples. The higher concentration of epsilon viniferin in the second sample can be attributed to its high solubility in polar solvents and its sensitivity to heat, resulting in more efficient extraction. Additionally, vacuum conditions were employed during the extraction process, as reducing pressure has been shown to increase mass transfer, further enhancing the efficiency of viniferin extraction (Khadhraoui et al., 2021).

Table 2 provides a comprehensive analysis of the four graphs obtained from the copper LC, offering valuable insights into the compounds present in the grape stem extracts and their relative concentrations under different extraction conditions. These data can serve as a foundation for further research and development of products that harness the potential health benefits of grape‐derived compounds.

## 4 CONCLUSION

This study employed a unique approach by combining three advanced techniques—maceration, microwave, and ultrasound—to break down plant cells and release hydrophilic and lipophilic polyphenols. It is worth noting that this method achieved these results without using nonpolar and toxic solvents, thereby improving efficiency and reducing extraction time. Additionally, a vacuum pump was integrated into the system for two specific samples to minimize thermal damage and separate oxygen molecules. As a result, this approach caused greater damage to the cell wall and enhanced nutrient release. Environmentally friendly solvents like water and a mixture of water and propylene glycol were used to extract the polyphenols.

The obtained extracts were analyzed using an LC–MS/MS device, which confirmed the presence of all expected polyphenolic compounds, except for luteolin, in high concentrations. The amount of each bioactive substance extracted varied depending on the compound's structural characteristics and the specific extraction method used. Therefore, the choice of extraction method can be customized to achieve the desired outcome in substance extraction.

Considering that compounds like quercetin, stilbene, procyanidins, resveratrol, and viniferin are primarily found in grapes and less commonly found in other agricultural waste materials, their highest concentrations were observed in samples 1 and 2. This can be attributed to the use of water as an economical and environmentally friendly solvent in these two methods. As a result, the maceration–microwave–ultrasound–water‐to‐water extraction method, with or without vacuum, is proposed as an innovative and health‐conscious approach for extracting polyphenols from grape stems that are typically discarded as waste in grape processing factories. These grape stems are a valuable source of bioactive compounds. The extracted polyphenols, such as quercetin, stilbene, procyanidins, resveratrol, and viniferin, hold promise for various applications in the food, pharmaceutical, and nutraceutical industries due to their antioxidant, anti‐inflammatory, and other bioactive properties.

In summary, the combination of these innovative techniques exhibits a synergistic effect that mitigates the drawbacks associated with individual methods. This approach effectively reduces extraction time and enhances the efficiency and quality of compounds sensitive to heat, oxygen, and light. Furthermore, it eliminates the need for organic solvents, overcoming the limitations of traditional methods. As a result, this method represents a promising new approach for extracting active compounds from plants. However, further optimization may be required to overcome certain limitations, such as the potential thermal damage caused by microwaves. Future studies should focus on investigating the bioactivities and potential health benefits of the identified polyphenols, as well as exploring additional green extraction methods and their applications in extracting valuable compounds from other agricultural waste materials.

## AUTHOR CONTRIBUTIONS

**Shiva Beilankouhi:** Conceptualization (equal); data curation (equal); investigation (equal); methodology (equal); software (equal); visualization (equal); writing – original draft (equal); writing – review and editing (equal). **Amir Pourfarzad:** Conceptualization (equal); data curation (equal); formal analysis (equal); funding acquisition (equal); investigation (equal); methodology (equal); project administration (equal); resources (equal); software (equal); supervision (equal); validation (equal); visualization (equal); writing – original draft (equal); writing – review and editing (equal). **Babak Ghanbarzadeh:** Supervision (equal); validation (equal). **Mousa Rasouli:** Funding acquisition (equal); investigation (equal); methodology (equal); resources (equal); validation (equal); visualization (equal). **Hamed Hamishekar:** Conceptualization (equal); investigation (equal); methodology (equal); software (equal); validation (equal).

## ACKNOWLEDGEMENTS

We gratefully acknowledge from Malayer University for supporting this project.

## FUNDING INFORMATION

This study was financially supported by the Malayer University, Iran with Grant number of 3‐1‐1‐4616.

## CONFLICT OF INTEREST STATEMENT

There are no known conflicts of interest that could have influenced the research presented in this manuscript.

## Data Availability

The data that support the findings of this study are available on request from the corresponding author. The data are not publicly available due to privacy or ethical restrictions.
